# Near infrared light fluorescence imaging-guided biomimetic nanoparticles of extracellular vesicles deliver indocyanine green and paclitaxel for hyperthermia combined with chemotherapy against glioma

**DOI:** 10.1186/s12951-021-00907-3

**Published:** 2021-07-14

**Authors:** Meng Wang, Chen-Yan Lv, Shu-Ang Li, Jun-Kuan Wang, Wen-Zheng Luo, Pei-Chao Zhao, Xue-You Liu, Ze-Ming Wang, Yang Jiao, Hong-Wei Sun, Yi Zhao, Peng Zhang

**Affiliations:** 1 Department of Neurosurgery, The First Affiliated Hospital of Zhengzhou University, No. 1, Jianshe East Road, Zhengzhou, 450052 Henan People’s Republic of China; 2 The Second Affiliated Hospital of Zhengzhou University, Zhengzhou, 450003 People’s Republic of China; 3 Clinical Systems Biology Laboratories, The First Affiliated Hospital of Zhengzhou University, Zhengzhou, 450052 People’s Republic of China; 4 Department of Translational Medicine Center, The First Affiliated Hospital of Zhengzhou University, No. 1, Jianshe East Road, Zhengzhou, 450052 Henan People’s Republic of China

**Keywords:** Glioma, Extracellular vesicles, Near infrared light, Indocyanine green, Paclitaxel, RGE, Hyperthermia, Chemotherapy

## Abstract

**Background:**

We investigated the therapeutic effect of targeting extracellular vesicles (EVs) loaded with indocyanine green (ICG) and paclitaxel (PTX) on glioma.

**Methods:**

Raw264.7 cells were harvested to extract EVs for the preparation of ICG/PTX@RGE-EV by electroporation and click chemistry. We evaluated the success of modifying Neuropilin-1 targeting peptide (RGE) on the EV membrane of ICG/PTX@RGE-EV using super-resolution fluorescence microscopy and flow cytometry. Spectrophotometry and high performance liquid chromatography (HPLC) were implemented for qualitative and quantitative analysis of the ICG and PTX loaded in EVs. Photothermal properties of the vesicles were evaluated by exposing to 808-nm laser light. Western blot analysis, cell counting kit 8 (CCK-8), Calcein Acetoxymethyl Ester/propidium iodide (Calcein-AM/PI) staining, and flow cytometry were utilized for assessing effects of vesicle treatment on cellular behaviors. A nude mouse model bearing glioma was established to test the targeting ability and anti-tumor action of ICG/PTX@RGE-EV in vivo.

**Results:**

Under exposure to 808-nm laser light, ICG/PTX@RGE-EV showed good photothermal properties and promotion of PTX release from EVs. ICG/PTX@RGE-EV effectively targeted U251 cells, with activation of the Caspase-3 pathway and elevated apoptosis in U251 cells through chemotherapy combined with hyperthermia. The anti-tumor function of ICG/PTX@RGE-EV was confirmed in the glioma mice via increased accumulation of PTX in the ICG/PTX@RGE-EV group and an increased median survival of 48 days in the ICG/PTX@RGE-EV group as compared to 25 days in the PBS group.

**Conclusion:**

ICG/PTX@RGE-EV might actively target glioma to repress tumor growth by accelerating glioma cell apoptosis through combined chemotherapy-hyperthermia.

**Graphic Abstract:**

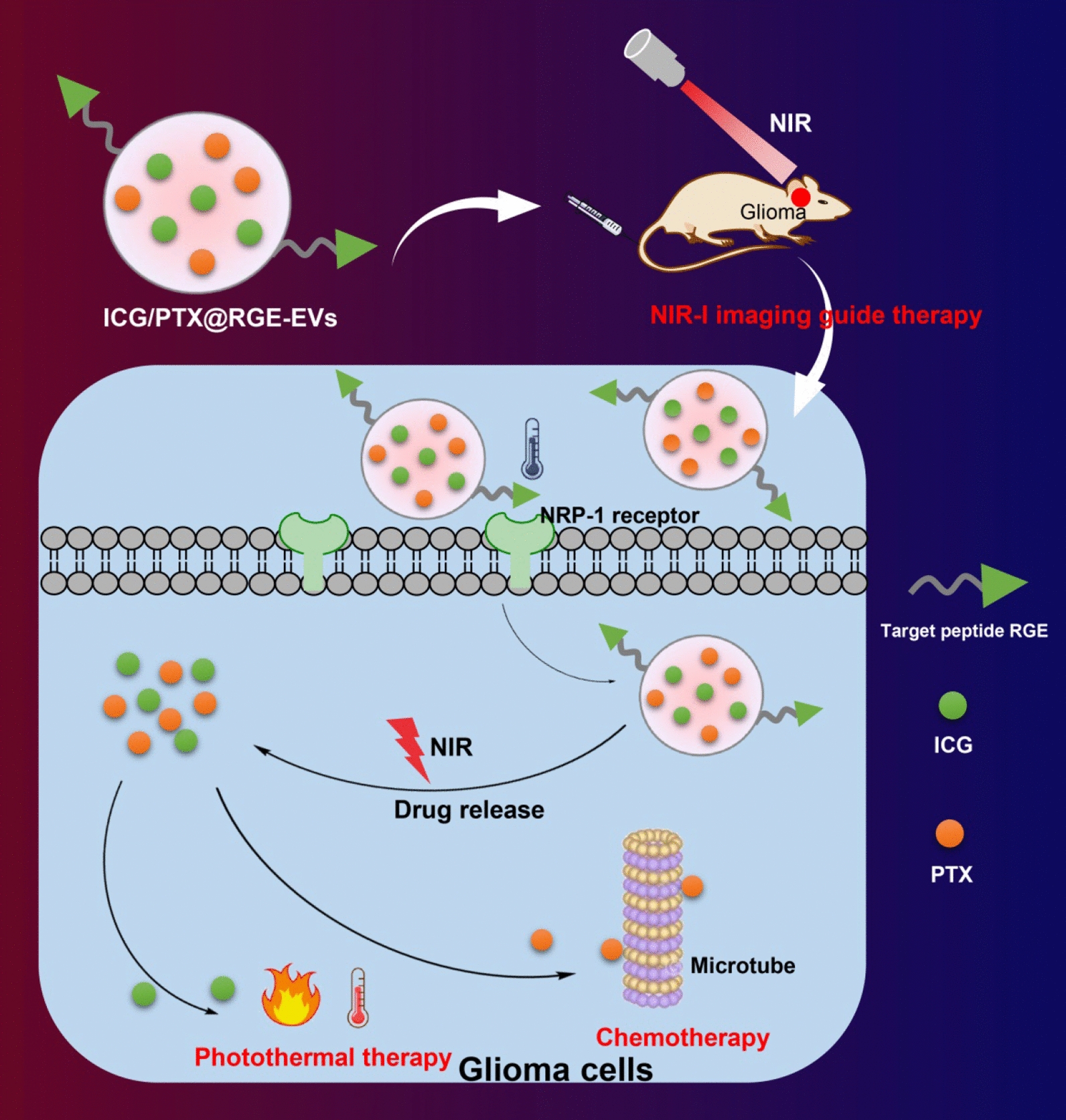

**Supplementary Information:**

The online version contains supplementary material available at 10.1186/s12951-021-00907-3.

## Introduction

Glioma, the most frequently occurring primary malignant malignancy in the human brain, remains as an incurable disorder despite currently available treatment protocols, including surgery, radiation, and chemotherapy [1]. Of note, increasing attention has been drawn in recent years to targeted therapy and combination therapy against glioma, yet there are few available reports regarding randomized controlled trials, literature reviews, basic medical research, and meta-analyses [2]. Chemotherapy has been employed in the management of almost all newly diagnosed diffuse gliomas to postpone the application of radiotherapy, suppress tumor growth, and improve survival and clinical symptoms of adult patients [3, 4]. To increase uptake of anticancer drugs, compromising the integrity of the glioma blood–brain barrier through hyperthermia triggered by water bath, radiofrequency microwaves, magnetic energy, laser-induced interstitial hyperthermia, and focused ultrasound, has emerged as a promising regimen for glioma treatment [5]. Hyperthermia, defined as a treatment protocol induced by abnormal fever, depends on temperature and exposure while precise targeting, accurate delivery and effective doses are required [6, 7]. More importantly, regional hyperthermia has been indicated to augment the efficacy of chemotherapy in the neoadjuvant first-line glioma therapy setting not only for children, but also for adolescents [8]. Therefore, we sought to investigate the therapeutic effect of chemotherapy combined with hyperthermia in managing glioma.

Extracellular vesicles (EVs), which are membranous nanoparticles carrying cargos of RNA, lipids, DNA, and protein, have been recognized to cross the blood–brain barrier in glioma [9]. As such, EVs are naturally produced nanoparticles that can function as drug delivery vehicles for medicine transport [10]. In addition, EV loading and surface modification play an important role in targeting the delivery of EVs encapsulated with therapeutic molecules or factors for the treatment of neurological disease [11]. Researchers have highlighted the huge potential of EVs in the therapy of various neoplasms, including glioma [12]. A previous report has elucidated that silk fibroin nanoparticles loaded with indocyanine green (ICG) in conjunction with near infrared (NIR) light for hyperthermia can kill residual glioma niche cells following surgical resection of the main tumor mass [13]. Paclitaxel (PTX), a chemotherapeutic agent, exhibits outstanding therapeutic efficacy for management of glioma, when used in conjunction with a functional drug delivery system to overcome the poor penetration across the blood brain barrier [14]. NIR-guided combined hyperthermia-chemotherapy, which selectively identifies cancer cells by enhancing visual differences between normal and tumor tissues [15], is becoming more prevalent in clinical practice. However, there remains limited research about whether NIR-guided biomimetic nanoparticles of EVS delivering ICG and PTX can orchestrate and potentiate the therapeutic role of hyperthermia combined with chemotherapy against glioma. Given the aforementioned evidence, we conducted the current study to explore the role of RGE modified EV co-delivery of PTX and ICG into glioma cells in the treatment of glioma through chemotherapy combined with hyperthermia.

## Results

### ICG/PTX@Neuropilin-1 targeting peptide (RGE)-EVs were successfully established

**Fig. 1:**
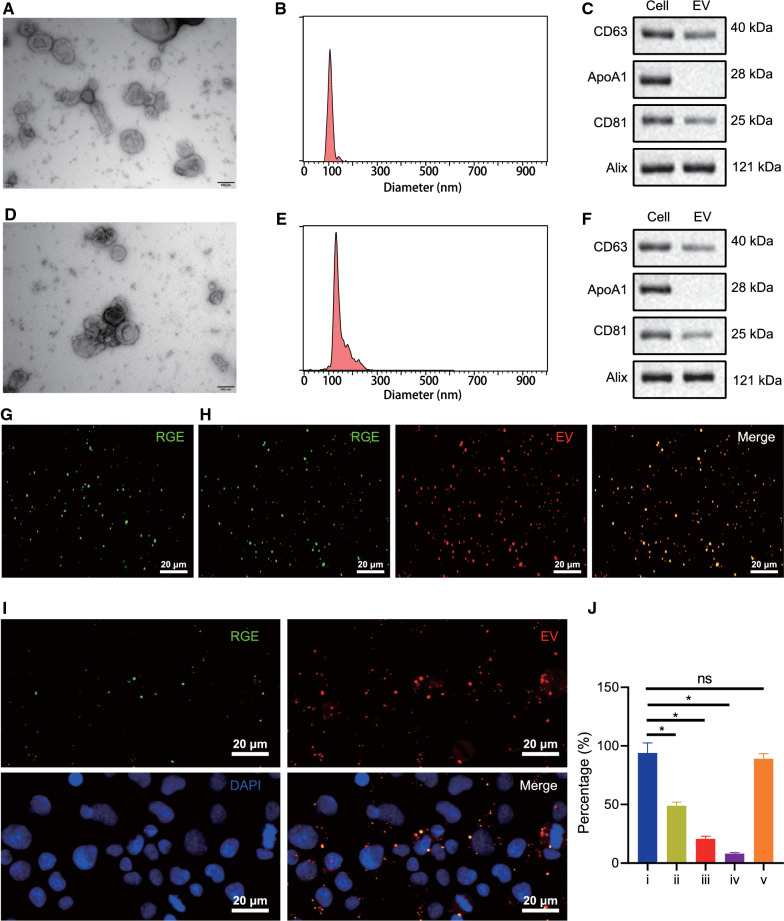
Preparation and identification of ICG/PTX@RGE-EV. **A** Morphological characteristics of EVs derived from Raw264.7 cells under TEM (scale bar = 100 nm). **B** Diameter of EVs detected by NTA. **C** Protein expression of EV markers, CD63, CD81, Alix and EV contamination index ApoA1 detected by Western blot analysis. **D** Morphological characteristics of ICG/PTX@RGE-EV under TEM (scale bar = 100 nm). **E** Diameter of EVs detected by NTA. **F** Protein expression of EV markers, CD63, CD81, Alix and EV contamination index ApoA1 in ICG/PTX@RGE-EV detected by Western blot analysis. **G** ICG/PTX@RGE-EV observed under super-resolution fluorescence microscopy (green fluorescence: RGE) (scale bar = 20 μm). **H** ICG/PTX@RGE-EV under confocal microscope (red fluorescence: EV, green fluorescence: RGE) (scale bar = 20 μm). **I** U251 cells incubated with CM-DiI-labeled ICG/PTX@RGE-EV observed under confocal microscope (scale bar = 20 μm). **J** FITC-positive cells identified by flow cytometry (i, U251 cells incubated with ICG/PTX@RGE-EV; ii, U251 cells incubated with ICG/PTX@ EV and RGE; iii, U251 cells incubated with ICG/PTX@EV; iv, free U251 cells; v, FITC-labeled U251 cells). Measurement data were expressed as mean ± standard deviation. Data comparison was analyzed by one-way ANOVA among multiple groups, followed by Tukey’s post hoc test. *p < 0.05 vs. the i group. The experiment was repeated in triplicate

To reduce effect on immune system, nude mouse Raw264.7 cells were used as parental cells, from which EVs were isolated through ultracentrifugation. Under transmission electron microscopy (TEM), we observed a group of heterogenous round/oval membranous vesicles with membranous protrusions (Fig. 1A). Nanoparticle tracking analysis (NTA) results revealed a mean diameter of 110.8 nm for the isolated EVs (Fig. 1B). Western blot analysis identified the expression of CD63, CD81 and Alix, which are all markers of EV membrane (Fig. 1C). These findings demonstrated that EVs were successfully isolated from Raw264.7 cells.

ICG/PTX@RGE-EV was prepared following methods described by Jia et al., followed by detection of their morphological characteristics [16]. TEM results revealed round ICG/PTX@RGE-EV of regular size (Fig. 1D), and NTA showed that the peak of ICG/PTX@RGE-EV size was 122.7 ± 6.5 nm (Fig. 1E). Also, ICG/PTX@RGE-EVs were found to be positive for CD63, CD81 and Alix, yet negative for the EV contamination index ApoA1, as detected by Western blot analysis (Fig. 1F; Additional file 1: Fig. S1). These results confirmed that ICG/PTX@RGE-EV maintained the integrity and characteristics of EVs.

To determine that EVs were successfully modified by RGE, purified ICG/PTX@RGE-EV suspension was added to the culture dish and RGE (green)-labeled round structures were observed under a super-resolution fluorescence microscope [where fluorescein isothiocyanate (FITC) indicates RGE] (Fig. 1G). Then, CM-DiI-labeled ICG/PTX@RGE-EVs were added dropwise onto the confocal microscope Petri dish and red fluorescence round structures were observed under the super-resolution fluorescence microscope, while red and green fluorescence were co-localized on the EV membranes (yellow color) (Fig. 1H). Following 2-h incubation of U251 cells with CM-DiI-labeled ICG/PTX@RGE-EV, the distribution of CM-DiI and FITC fluorescence was observed under the confocal microscope, results of which revealed that EV membranes (red fluorescence) largely overlapped with RGE (green fluorescence) in U251 cells (Fig. 1I). Further flow cytometric analysis for the detection of FITC-positive cells revealed a similar percentage of FITC-positive cells in U251 cells co-incubated with ICG/PTX@RGE-EV to that in FITC-labeled cells, which was significantly higher than in control cells (Fig. 1J). Taken together, we found ICG/PTX@RGE-EVs were successfully modified by RGE-peptide.

### ICG and PTX were loaded onto ICG/PTX@RGE-EV

**Fig. 2:**
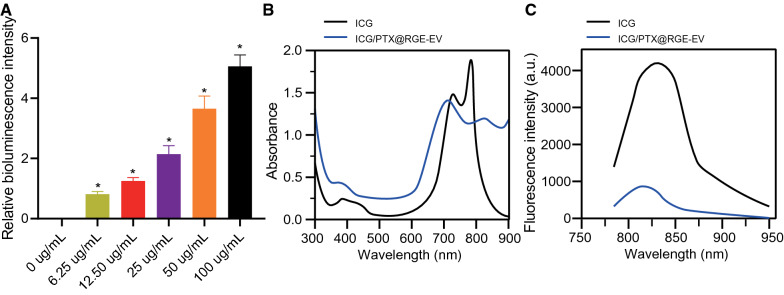
Uptake and emission of ICG/PTX@RGE-EV. **A** Fluorescence intensity of NIR fluorescence imaging in ICG/PTX@RGE-EV of different concentrations. **B** UV–VIS absorption spectra of free ICG and ICG/PTX@RGE-EV. **C** Fluorescence emission spectra of free ICG and ICG/PTX@RGE-EV (excitation wavelength: 808 nm). Measurement data were expressed as mean ± standard deviation. Data comparison was analyzed by one-way ANOVA among multiple groups, followed by Tukey’s post hoc test. *p < 0.05 vs*.* the 0 µg/mL group. The experiment was repeated three times independently

Subsequently, ultraviolet (UV) and fluorescence spectrum analysis and high performance liquid chromatography (HPLC) were performed to identify whether EVs were successfully loaded with ICG and PTX. NIR fluorescence imaging results revealed increasingly enhanced visualized fluorescence signal with increasing ICG/PTX@RGE-EV concentration (Fig. 2A). Ultraviolet–visible spectroscopy (UV–VIS) spectroscopy and fluorescence emission spectrum results showed a relatively wide absorption peak between 600 and 800 nm of free ICG and ICG/PTX@RGE-EV, while the fluorescence spectrum revealed two characteristic peaks between 780 and 850 nm, although the absorption peak and fluorescence emission peak were reduced relative to that of free ICG (Fig. 2B, C). We attributed this reduced fluorescence intensity to the rapid quenching of ICG in EVs under strong laser irradiation. The aforementioned results suggested that ICG was successfully loaded in EVs. Then, HPLC analysis provided further evidence confirming that PTX was successfully loaded in EVs. The concentrations of RGE, ICG and PTX were measured (Additional file 2: Fig. S2A–C), results of which revealed high loading of modified RGE, ICG and PTX in ICG/PTX@RGE-EV, thus ensuring targeting ability and applicability for subsequent experiments.

### ICG/PTX@RGE-EV was featured with photothermal properties to promote PTX release

**Fig. 3:**
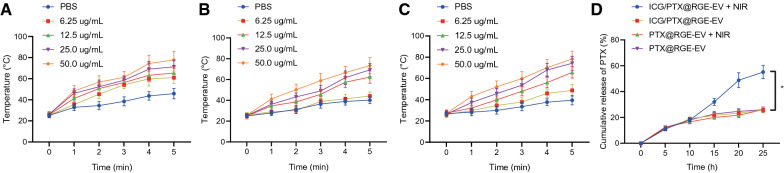
ICG/PTX@RGE-EV featured with photothermal properties contributes to PTX release. **A** Temperature of ICG at different concentrations under exposure to 1.25 W/cm^2^ laser. **B** Temperature of ICG/PTX@EV at different concentrations under exposure to 1.25 W/cm^2^ laser light. **C** Temperature of ICG/PTX@RGE-EV at different concentrations under exposure to 1.25 W/cm^2^ laser light. **D** Cumulative release curve of PTX after ICG/PTX@RGE-EV and PTX@RGE-EV were stirred at an initial temperature of 37 °C at 100 rpm. The ICG/PTX@RGE-EV + NIR and PTX@RGE-EV + NIR groups were irradiated with NIR light (808 nm, 1.0 W/cm^2^) for 5 min at predetermined time points (10, 15, 20, and 24 h). PTX concentration was determined by HPLC. Measurement data were expressed as mean ± standard deviation. Data comparison was analyzed by one-way ANOVA among multiple groups, followed by Tukey’s post hoc test. *p < 0.05 vs. the ICG/PTX@RGE-EV + NIR group. The experiment was repeated three times independently

The aim of the subsequent experiments was to evaluate photothermal effects of ICG/PTX@RGE-EV in vitro. The UV–VIS absorption spectra of ICG/PTX@RGE-EV showed a strong near infrared absorption at 808 nm, so we chose to use a laser tuned at 808 nm to irradiate solutions of different concentrations and then record their temperature by thermal imaging every 10 s for 5 min in total. As revealed the from time–temperature curve, temperature increased with higher ICG/PTX@RGE-EV concentration, reaching approximately 60 °C for a concentration of 50 μg/mL, while the temperature remained unchanged in a medium containing only phosphate buffered saline (PBS) (Fig. 3A–C). Additionally, solution at a fixed concentration was exposed to laser of different powers; results showed that the temperature increased when laser power was elevated from 0.5 W/cm^2^ to 1.5 W/cm^2^ (Additional file 3: Fig. S3). By comparing temperature changes of ICG, ICG/PTX@EV and ICG/PTX@RGE-EV, we found that temperature of ICG/PTX@RGE-EV following laser irradiation did not differ significantly in presence of EV entrapment and RGE-peptide modification. These results indicated that ICG/PTX@RGE-EV had appropriate photothermal properties for photothermal therapy.

Then, the effect of NIR light on PTX release was detected by dynamic dialysis. This showed that PTX release from ICG/PTX@RGE-EV and PTX@RGE-EV remained slow before exposure to NIR light, but NIR illumination significantly accelerated the release of PTX from ICG/PTX@RGE-EV, while having no such effect with PTX@RGE-EV. At 24 h after the last NIR light exposure, the cumulative release of PTX reached approximately 55% from ICG/PTX@RGE-EV, yet only 25% from ICG/PTX@RGE-EV without NIR light. Furthermore, there was no significant difference regarding PTX release from EVs under NIR in the absence of ICG (Fig. 3D). To conclude, these findings demonstrated that NIR light activated ICG to release heat as a contributing factor in the augmented release of PTX.

### ICG/PTX@RGE-EV effectively targeted U251 cells in vitro

**Fig. 4:**
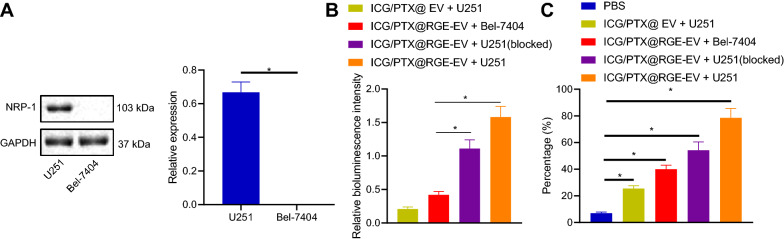
Uptake and targeting abilities of ICG/PTX@RGE-EV were identified in vitro. **A** Expression level of NRP-1 in U251 and Bel-7404 cells detected by Western blot analysis. **B** Fluorescence intensity of ICG in different groups. **C** ICG-positive cell percentage detected by flow cytometry. Measurement data were expressed as mean ± standard deviation. Data comparison was analyzed by one-way ANOVA among multiple groups, followed by Tukey’s post hoc test. *p < 0.05. The experiment was repeated 3 times independently

Neuropilin-1 (NRP-1) is highly expressed in multiple tumors, including breast cancer, prostate cancer, colon cancer and glioma. Therefore, the specific targeting property of RGE-peptide on NRP-1 was applied for active targeting in glioma. Western blot analysis revealed upregulated NRP-1 expression in U251 cells yet NRP-1 expression was barely detectable in Bel-7404 cells (Fig. 4A). Therefore, Bel-7404 cells were used as controls in subsequent studies. ICG/PTX@RGE-EV was incubated with U251 (NRP-1-positive) or Bel-7404 (NRP-1-negative) cells for 4 h. As revealed from ICG fluorescence imaging, the fluorescence signal was the strongest in U251 cells incubated with ICG/PTX@RGE-EV, yet significantly reduced in U251 cells blocked with RGE-peptide (1 nmol/10^5^ cells) (Fig. 4B). Flow cytometric analysis revealed highest percentage of ICG-positive cells for U251 cells incubated with ICG/PTX@RGE-EV, followed by U251 cells blocked with RGE in the presence of ICG/PTX@RGE-EV, whereas few positive cells were detected among U251 cells incubated with ICG/PTX@EV (Fig. 4C). These results revealed the efficient targeting ability of ICG/PTX@RGE-EV on U251 cells in vitro.

### ICG/PTX@RGE-EV induced U251 cell apoptosis by activating the Caspase-3 pathway through chemotherapy combined with hyperthermia

**Fig. 5:**
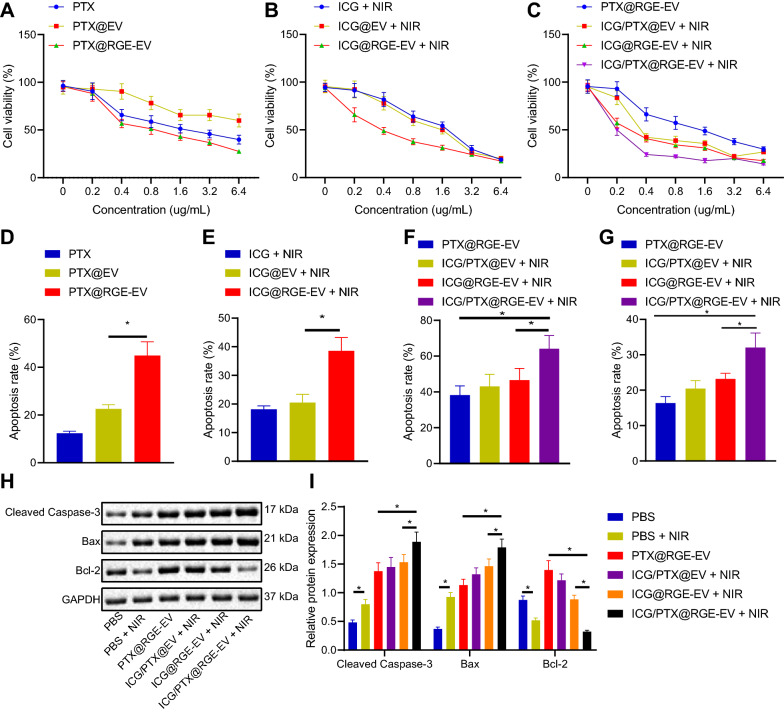
ICG/PTX@RGE-EV contributes to apoptosis of U251 cells. **A** Cell viability following chemotherapy detected by CCK8 assay (x-axis represents PTX concentration, which was set to 0–6.4 µg/mL). **B** Cell viability following hyperthermia detected by CCK8 assay (x-axis represents ICG concentration, which was set to 0–6.4 µg/mL). **C** Cell viability following chemotherapy-hyperthermia detected by CCK8 assay (x-axis represents PTX concentration, which was set to 0–6.4 µg/mL). U251 cells were incubated with PBS, RGE-EV, or PTX@RGE-EV for 48 h, respectively, or incubated with ICG@RGE-EV or ICG/PTX@RGE-EV for 4 h and then irradiated with 808 nm laser light (0.8 W/cm^2^) for five min, followed by incubation for 44 h. Each group was calculated by PTX concentration (0.2 µg/mL) in panel **D**–**F**. **D** Cell apoptosis identified by Calcein AM/PI staining assay following chemotherapy. **E** Cell apoptosis identified by Calcein AM/PI staining assay following hyperthermia. **F** Cell apoptosis identified by Calcein AM/PI staining assay following chemotherapy-hyperthermia. **G** Cell apoptosis rates determined by flow cytometry. Each group was calculated by PTX concentration (0.2 µg/mL). **H** Protein bands of Cleaved Caspase-3, Bax and Bcl-2 detected by Western blot analysis. **I** Relative protein expression of Cleaved Caspase-3, Bax and Bcl-2 detected by Western blot analysis. Measurement data were expressed as mean ± standard deviation. Data comparison was analyzed by one-way ANOVA among multiple groups, followed by Tukey’s post hoc test. *p < 0.05. The experiment was repeated three times independently

Subsequent experiments were performed to explore the killing effect on tumor cells. U251 cell viability and dead cells were detected by Cell counting kit 8 (CCK-8) and Calcein Acetoxymethyl Ester/propidium iodide (Calcein AM/PI) staining assays. Following chemotherapy, RGE-modified EVs loaded with PTX significantly promoted the killing effect on tumor cells accompanied by larger staining area of dead cells than seen with PTX@EV (Fig. 5A, D). Following hyperthermia, the killing effect on tumor cells was significantly enhanced by treatment with RGE-modified EVs loaded with ICG, accompanied by a larger staining area of dead cells under laser irradiation than with ICG and ICG@EV (Fig. 5B, E). In cells treated with chemotherapy-hyperthermia (ICG/PTX@RGE-EV), a stronger killing effect on tumor cells accompanied by larger staining area of dead cells was found when compared with chemotherapy or hyperthermia treatment alone (Fig. 5C, F). The aforementioned findings indicated that ICG/PTX-loaded EVs significantly promoted the killing effect on tumor cells following RGE modification in comparison to results seen with free ICG or PTX. Also, chemotherapy combined with hyperthermia further potentiated the anti-tumor action in comparison to single therapy.

Furthermore, cell apoptosis was analyzed following combined treatment of chemotherapy and hyperthermia. Flow cytometric analysis, RT-qPCR and Western blot analysis revealed the highest apoptosis rate in cells treated with RGE-modified chemotherapy-hyperthermia, which was accompanied by highest expression of Cleaved Caspase-3 and Bax, as well as the lowest Bcl-2 expression (Fig. 5G–I). Moreover, the apoptosis rate following chemotherapy-hyperthermia without RGE modification ranked between that of chemotherapy and hyperthermia alone (Fig. 5G). Taken together, these findings demonstrated that ICG/PTX@RGE-EV triggered apoptosis of U251 cells through Caspase-3 activation following the combined treatment of chemotherapy and hyperthermia.

### ICG/PTX@RGE-EV effectively targeted glioma in vivo

**Fig. 6:**
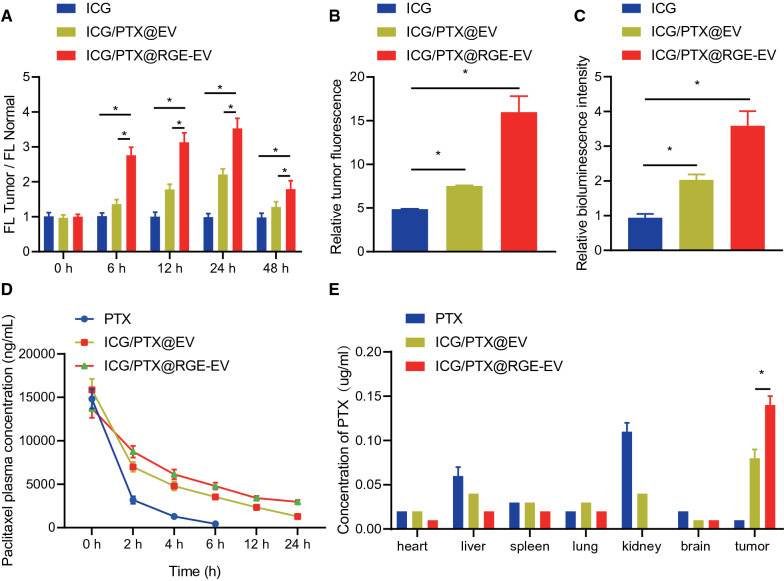
Targeting effect of ICG/PTX@RGE-EV on glioma was identified in vivo. **A** Fluorescence intensity ratio of tumor and normal tissues of nude mice following different treatments at different time points. **B** The relative statistical fluorescence imaging of tumor tissues of nude mice. **C** Fluorescence intensity ratio of tumor sections from nude mice following different treatment. **D** PTX concentration distribution in the plasma of nude mice following different treatment. **E** PTX contents in the heart, liver, spleen, lung, kidney, brain and tumor a 24 h after administration of nude mice determined with HPLC. Measurement data were expressed as mean ± standard deviation. Data comparison was analyzed by one-way ANOVA among multiple groups, followed by Tukey’s post hoc test. Data at different time points were compared by repeated measures ANOVA among multiple groups, followed by Tukey’s post hoc test. *p < 0.05. The experiment was repeated three times independently

For further investigation regarding the targeting effect of ICG/PTX@RGE-EV on glioma in vivo, we developed a nude mouse model of glioma, with subsequent treatments by intravenous injection of ICG, ICG/PTX@EV and ICG/PTX@RGE-EV. Fluorescence imaging results revealed an extremely weak fluorescence signal in the brain of nude mice treated with ICG, indicating that ICG was not enriched in the brain. As for nude mice treated with ICG/PTX@EV, a relatively weak signal was detected at 12 h, which increased at 24 h but disappeared at 48 h, suggesting that ICG was rapidly metabolized and cleared from healthy brain tissue. Following treatment of ICG/PTX@RGE-EV, relatively strong fluorescence signal was detected at 6 h with extremely low background signal, which increased over time to a peak at 24 h. At the 48-h time-point, NIR fluorescence signal was also observed at the tumor site, suggesting that RGE-modified ICG/PTX@RGE-EV increased the accumulation period in the tumor. Additionally, the signal-to-background ratio at all imaging time points was highest in the ICG/PTX@RGE-EV group when compared with the ICG and ICG/PTX@EV groups (Fig. 6A).

To further verify that fluorescence signal in the brain was localized specifically at the tumor site, major organs (heart, liver, spleen, lung and kidney) and tumor tissues were collected from tumor-bearing mice for fluorescence imaging. Results revealed no significant difference in fluorescence signal between tumor tissues and major organs (heart, liver, spleen, lung, kidney and brain) of nude mice treated with ICG. The fluorescence signal at the tumor site of nude mice injected with ICG/PTX@EV was slightly stronger than that detected at the major organs, yet tumor tissues of nude mice injected with ICG/PTX@RGE-EV exhibited significantly stronger fluorescence signal in comparison to major organs. By comparing the relative fluorescence signal intensity (The relative fluorescence intensity was calculated by dividing the fluorescence intensity of tumor tissues in each group by the sum of the fluorescence intensity of the heart, liver, spleen, lung, kidney and brain) of tumor tissues in different groups, we found that the tumor fluorescence signal of ICG/PTX@RGE-EV was significantly stronger than that of ICG/PTX@EV (Fig. 6B). Fluorescence imaging on tumor sections revealed stronger fluorescence signal of ICG in and around the tumor of tumor-bearing nude mice treated with ICG/PTX@EV when compared with those treated with ICG, while distinctly stronger fluorescence signal was detected in the tumors following treatment of ICG/PTX@RGE-EV than in those following treatment of ICG/PTX@EV (Fig. 6C).

Subsequently, PTX content in tumor tissues was determined by HPLC at different time points to further document the targeting ability of ICG/PTX@RGE-EV by HPLC in presence of PTX, ICG/PTX@EV and ICG/PTX@RGE-EV. We showed that blood circulation time did not significantly differ between ICG/PTX@EV treatment and ICG/PTX@RGE-EV treatment. At 24 h, the PTX content in blood was still maintained. However, a significant reduction of the PTX content in blood was observed in the PTX group at the 6 h time point, suggesting that EVs had significantly increased the accumulation of PTX as vectors amenable to crossing the blood–brain barrier (Fig. 6D). The mice were euthanized 24 h after the treatment, and the major organs and tumor were removed. HPLC analysis revealed high PTX concentrations in the liver and kidney but the lowest PTX at the tumor site. Accumulation of PTX was significantly higher in the tumor tissues of the ICG/PTX@EV and ICG/PTX@RGE-EV groups than that in the normal tissues, while more PTX was detected in the tumor site upon treatment with ICG/PTX@RGE-EV compared with ICG/PTX@EV (Fig. 6E). Collectively, the experimental data indicated efficient targeting ability of ICG/PTX@RGE-EV on glioma in vivo.

### ICG/PTX@RGE-EV prolonged survival period of mice with glioma by inhibiting tumor growth through chemotherapy-hyperthermia

Finally, tumor-bearing mice were treated with PBS, PBS + NIR, PTX@RGE-EV, ICG@RGE-EV + NIR, ICG/PTX@EV + NIR or ICG/PTX@RGE-EV + NIR. The dose of each mouse was equivalent to 10 mg/kg PTX of body weight. As shown in infrared thermal image, after 5 min of irradiation the brain temperature was 38 °C in mice treated with PBS + NIR, 43 °C in mice treated with ICG@RGE-EV + NIR, and 42.2 °C in mice treated with ICG/PTX@EV + NIR, while the highest of 48 °C found in mice following combined therapy (ICG/PTX@RGE-EV + NIR). Bioluminescence images revealed the strongest anti-tumor action following targeting combined therapy (ICG/PTX@RGE-EV + NIR), which was significantly better than for chemotherapy (PTX@RGE-EV), hyperthermia (ICG@RGE-EV + NIR), and non-targeted chemotherapy-hyperthermia (ICG/PTX@EV + NIR).

**Fig. 7:**
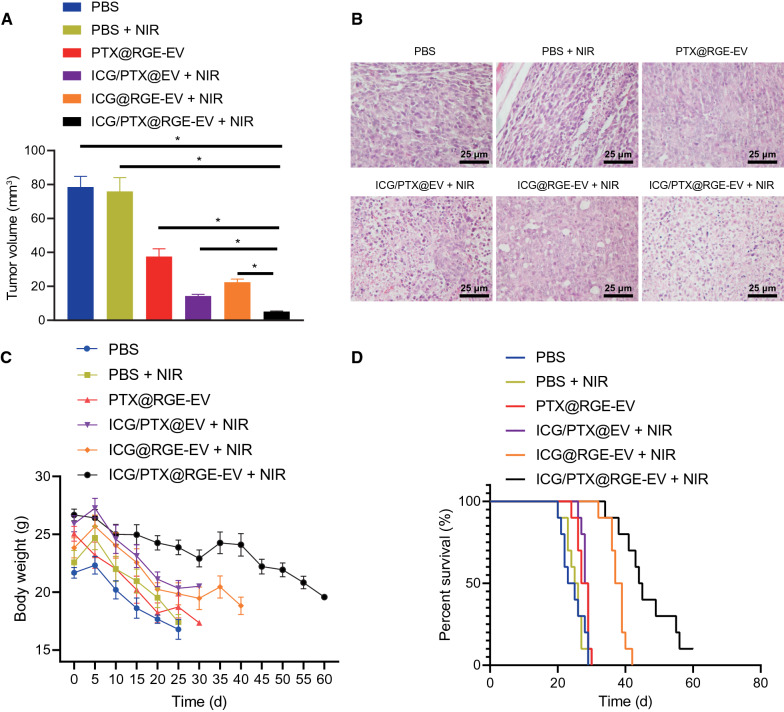
ICG/PTX@RGE-EV exerts inhibitory effect on glioma growth in vivo. **A** Tumor volume. **B** Tumor tissues identified by HE staining. **C** Nude mouse weight. **D** Survival period and median survival of nude mice (n = 10 mice/each group). Measurement data were expressed as mean ± standard deviation. Data comparison was analyzed by one-way ANOVA among multiple groups, followed by Tukey’s post hoc test. Data at different time points were compared by repeated measures ANOVA among multiple groups, followed by Tukey’s post hoc test. Survival rate of nude mice was calculated by the Kaplan–Meier plot. *p < 0.05. The experiment was repeated three times independently. ICG/PTX@RGE-EV + NIR: targeting combined therapy, PTX@RGE-EV: chemotherapy; ICG@RGE-EV + NIR: hyperthermia; ICG/PTX@EV + NIR: non-targeted chemotherapy-hyperthermia

Significantly reduced tumor volume was observed in mice following targeting combined therapy (ICG/PTX@RGE-EV + NIR) compared with mice treated with chemotherapy, (PTX@RGE-EV), hyperthermia (ICG@RGE-EV + NIR), or non-targeted chemotherapy-hyperthermia (ICG/PTX@EV + NIR) (Fig. 7A). Moreover, tumor cells were tightly arranged with large nuclei and few cytoplasm characterized by obvious cellular atypia in mice treated with PBS or PBS + NIR. Following chemotherapy (PTX@RGE-EV), hyperthermia (ICG@RGE-EV + NIR), non-targeted chemotherapy-hyperthermia (ICG/PTX@EV + NIR), and targeted chemotherapy-hyperthermia (ICG/PTX@RGE-EV + NIR), tumor cells were sparser and replaced by fibrous tissues, especially following targeted chemotherapy-hyperthermia (ICG/PTX@RGE-EV + NIR) (Fig. 7B). TUNEL staining showed fewer green fluorescent cells in the PBS and PBS + NIR groups and greater numbers in the other groups, especially in the ICG/PTX@RGE-EV + NIR group (Additional file 4: Fig. S4). These results further elucidated the anti-tumor action of ICG/PTX@RGE-EV + NIR.

During the period under observation after therapy, mouse body weight had decreased the most on day 15 in the PBS and PBS + NIR groups, and the least weight loss was seen in mice treated with targeted chemotherapy-hyperthermia (ICG/PTX@RGE-EV + NIR) (Fig. 7C). Median survival of nude mice was 48 days following targeted combined therapy (ICG/PTX@RGE-EV + NIR), 30 days following chemotherapy (PTX@RGE-EV), 36 days following hyperthermia (ICG@RGE-EV + NIR), 33 days following non-targeted combined therapy (ICG/PTX@EV + NIR), 25 days following PBS treatment, and 26 days following treatment with PBS + NIR (Fig. 7D). In conclusion, targeted combined therapy significantly suppressed tumor growth while reducing toxic effect on major organs to prolong survival period of nude mice.

### Determination of RGE, ICG and PTX concentrations

First, we plotted an absorbance-concentration of RGE-peptide (FITC) standard curve (Additional file 1: Fig. S1A). Based on the absorbance of RGE-FITC in ICG/PTX@RGE-EV, the EVs-modified RGE-peptide concentration was 98 nM. Combined with the number of particles detected by NTA, we thus estimate that a mean of 52 EVs were binding to RGE-peptide. According to standard curves of the absorbance-concentration of ICG and HPLC peak area-concentration of PTX (Additional file 1: Fig. S1B, C), ICG and PTX concentrations were 4000 µg ± 20 µg/mL trehalose PBS and 4000 µg ± 35 µg/mL trehalose PBS, respectively, indicating entrapment efficiencies of 90 and 93%, respectively. These findings indicated the relatively higher loading efficiency of RGE, ICG and PTX in ICG/PTX@RGE-EV, which met the need of targeting ability and practicability for subsequent experiments.

### ICG/PTX@RGE-EV stability in vitro

The obtained ICG/PTX@RGE-EV was divided into 12 aliquots and stored at − 80 °C. The contents of RGE, ICG and PTX in the suspension were determined on a weekly basis to assess stability. TEM and NTA were performed at the 4th week, followed by Western blot analysis on CD63 and CD81. According to fluorescence intensity of RGE-FITC and ICG and the HPLC peak area measurements, the concentrations of RGE, ICG and PTX did not change significantly in the suspension with storage time (Additional file 5: Fig. S5A–C), yet expression of CD63, CD81 and EV contamination index ApoA1 slightly increased in the suspension when compared with Raw264.7 cells (Additional file 5: Fig. S5D). The mean particle size was stabilized at 120 nm, indicating the integrity of ICG/PTX@RGE-EV (Additional file 5: Fig. S5E). These results highlighted the stability of ICG/PTX@RGE-EV to long term storage.

## Discussion

As the most aggressive and malignant tumor occurring in the brain, glioma is responsible for an enormous number of deaths in cases related to brain tumors around the world [17]. The promising advantages of low immunogenicity, intrinsic cell targeting properties, and enhanced stability in circulation make EVs an ever more attractive concept in targeted drug therapy, and highlight their potential in the therapy of various diseases, including glioma [12]. Recently, cell penetrating peptides have emerged as a critical player in targeted therapy for glioma by embellishing nanocarriers to deliver anti-tumor drugs [18]. During the current investigation, we aimed to explore the therapeutic effect of ICG/PTX transferred by RGE-EV in glioma. Collectively, the experimental data demonstrated that ICG/PTX@RGE-EV contributed to apoptosis of malignant cells by activating the Caspase-3 pathway through chemotherapy-hyperthermia, with the favorable results that glioma progression was suppressed in vitro and in vivo.

Following successful establishment of ICG/PTX@RGE-EV, which were characterized by good photothermal properties, the release of PTX from ICG/PTX@RGE-EV was promoted by exposure to NIR light, in a manner augmented by the heat released from ICG activation. PTX is known as a microtubule stabilizing drug for treating glioma, yet its efficacy remains suboptimal because of its poor penetration across the blood–brain barrier [19]. Intriguingly, a prior research revealed that NIR light induced the release of PTX from biodegradable microspheres to enhance the photothermal effect and antitumor activity [20]. ICG is an NIR dye approved by the Food and Drug Administration (FDA), which serves at once as a photothermal and photodynamic agent [21]. Largely in agreement with our present findings, NIR has been elucidated to exert photothermal effects through a co-release system of the chemotherapeutic agent doxorubicin and the photosensitizer ICG, thereby functioning as a chemo/photothermal/photodynamic treatment against multidrug resistant human breast cancer [22]. Besides, EVs can also be utilized in photothermal therapy [23]. PTX- and doxorubicin-loaded nanostructured lipid carriers have been reported to exert an inhibitory function on the growth of glioma stem cells [24]. Of note, PTX encapsulated in EVs has been indicated to have a higher transduction ratio and promoted the anti-tumor action against lung cancer as compared to PTX alone in vitro and in vivo [25]. More importantly, our present results manifested that ICG/PTX@RGE-EV effectively targeted glioma in vivo and in vitro. Furthermore, bioluminescence and fluorescence imaging performed in a previous study confirmed the targeting ability of EVs-loaded PTX in neoplasia without adverse effects on other body tissues, which is highly suggestive of the therapeutic promise of systemic administration via EVs combined with chemotherapy agents [26]. ICG has been utilized in researches of the photothermal treatment of glioma [12]. In addition, ultrasound imaging-guided chemotherapy-hyperthermia induced by NIR light has been identified as a promising therapeutic modality against malignancies [27].

Furthermore, in the present study, the combined treatment of chemotherapy-hyperthermia caused an upregulation of Cleaved Caspase-3 and Bax while downregulating Bcl-2, in contribution to glioma cell apoptosis, which was further enhanced by RGE embellishment. Consistent with this picture, nude mice following therapy of ICG/PTX@RGE-EV + NIR exhibited accelerated glioma cell apoptosis and reduced toxic effects on the body, along with increased survival period. Reduced Bcl-2 expression and elevated expression of Caspase-3 and Bax have been used previously as indicators of induced apoptosis of glioma cells and desirable oncologic outcomes of patients with glioma following radiotherapy and/or chemotherapy [28–30]. Caspase-3 activation has been widely recognized as a factor in the therapy of various cancers including glioma, yet Caspase-3 activation in dying glioma cells has been noted to create an unfavorable environment for angiogenesis after irradiation [31, 32], suggesting the need of more in-depth studies. Concordant with our present results, a prior work indicated that ICG-based therapy could trigger increased cell apoptosis in three cancer cell lines [33]. Also, PTX was capable of activating caspase-3 (the effector of apoptosis pathways) and reducing Bcl-2 expression (the anti-apoptotic protein) to repress glioma cell apoptosis [34], which was partially consistent with our findings. Moreover, nanobubbles carrying ICG and PTX cause a more significant promotion of prostate cancer cell apoptosis compared to treatments with PTX or ICG alone [35]. More importantly, NRP-1-targeted RGE-conjugated EVs have been documented to facilitate diagnosis and therapy of glioma, both in vitro and in vivo [16], which is in line with our finding that active targeting of ICG/PTX@RGE-EV in glioma was achieved by RGE-peptide specific targeting of NRP-1. The results of our study first elucidated that the inhibitory impact of ICG/PTX@RGE-EV on glioma development via apoptosis promotion, which suggested that ICG/PTX@RGE-EV might eventually be employed clinically for the treatment of glioma.

## Conclusions

**Fig. 8:**
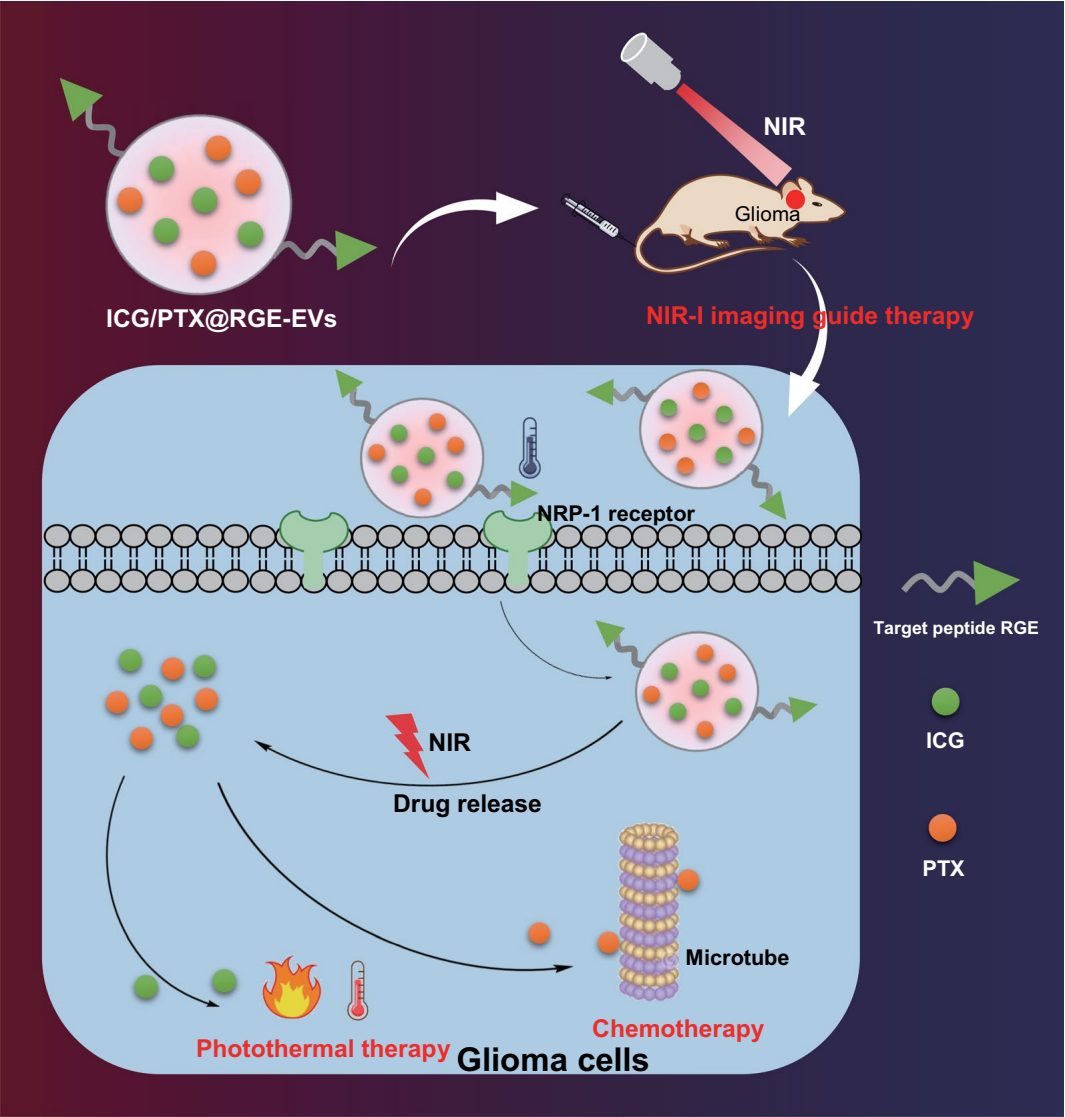
The graphical summary of the function and mechanism of ICG/PTX@RGE-EV in glioma. ICG/PTX@RGE-EV can actively target glioma to activate the Caspase-3 pathway through chemotherapy-hyperthermia in contribution to glioma cell apoptosis, by which means tumor growth is suppressed

To conclude, we report in the present study that ICG/PTX@RGE-EV can function as a potential drug delivery system of ICG/PTX for treatment of glioma, as evidenced by its prolonged residence period in blood circulation, and increased accumulation in tumor, thereby promoting anti-tumor action and reducing toxicity (Fig. 8). However, there are a number of limitations in our research. First, the mouse sample size was small, and much remained to be established before this approach with ICG/PTX@RGE-EV can ever achieve clinical translation. Just for example, we would need to produce GMP standard ICG/PTX@RGE-EV on a large scale, establish the safety of ICG/PTX@RGE-EV in humans, and learn how to accommodate individual differences in responses. Although there are significant challenges and difficulties in the application of EV-based drug delivery, this endogenous vesicle shows great potential in the biomedical field as the next generation of nanomaterials for advanced drug delivery and treatment.

## Materials and methods

### Experiment materials

Macrophages Raw264.7, human glioma cells U251 and human liver cancer cells Bel-7404 were purchased from Cell Bank, Chinese Academy of Sciences (Shanghai, China) and used for in vitro assays. Cells were cultured in Dulbecco’s Modified Eagle Medium containing 10% fetal bovine serum (FBS, 10099141, Gibco, Carlsbad, CA, USA) and antibiotic (Gibco) with 5% CO_2_ at 37 °C. The medium and reagent were provided by Gibco. ICG, Avanti and PTX were purchased from Yew Pharmaceutical Co., Ltd. (Hainan, China). Ultrafiltration centrifuge tubes (100 KDa) and polyvinylidene fluoride (PVDF) membranes were from Millipore (Billerica, MA, USA). RGE-peptide conjugated with FITC was purchased from KareBay Biochem (Ningbo, China). Antibodies to β-Actin, B-cell lymphoma-2 (Bcl-2), and Bcl-2 associated protein X were provided by Cell Signaling Technology (CST) (Danvers, MA, USA). Cell counting kit 8 (CCK8) and Calcein-AM/PI double staining kits were purchased from Dojindo (Japan), while the Annexin V-APC/7-amino-actinomycin D (7-AAD) apoptosis detection kit was bought from KeyGEN Biotech (Nanjing, China).

### Isolation and purification of EVs

Before culture, EVs were depleted through centrifugation at 120,000×*g* for 18 h using FBS. Raw264.7 cells were cultured with 10% EVs-depleted FBS. Approximately 1000 mL supernatant (5 × 10^8^ cells for 48-h culture) was subjected to centrifugation in a 100 KDa ultrafiltration centrifuge tube. The retentate was re-suspended in 100 mL PBS, centrifuged at 300×*g* for 10 min at 4 °C, at 2000×*g* for 10 min, and at 10,000×*g* for 30 min to remove cells and debris. The supernatant was filtered through 0.22 µm filter, followed by centrifugation in Ti70 rotor of L-80XP ultracentrifuge (Beckman Coulter, Brea, CA, USA) at 120,000×*g* for 70 min at 4 °C. The pelleted EVs were re-suspended in PBS and ultra-centrifuged at 120,000×*g* for 70 min. The EVs were again re-suspended in PBS and stored at − 80 °C for further use. The protein concentration in EVs was measured using the bicinchoninic acid (BCA) protein detection kit (Thermo Fisher Scientific, Santa Clara, CA, USA).

### Preparation of ICG/PTX@EV

When ICG, PTX and EV in the reaction system reached 1: 1: 1, weight ratio of ICG and PTX was 1: 1 in synthetized EVs, creating a favorable experimental condition for subsequent experiment. Therefore, 1 mL of trehalose PBS, containing 1000 μg EVs, 1000 μg PTX, and 1000 μg ICG, was electroporated in 4-mm path length electroporation cuvettes using a Bio-Rad electroporation instrument (400 V, 150 µF for 1 ms). The same experimental steps were performed in 1 mL of trehalose PBS, containing 1000 µg EVs and 1000 µg PTX as well as in 1 mL of trehalose PBS, containing 1000 µg EVs and 1000 µg ICG. One pulse was applied to each EV sample under the conditions as mentioned above. After that, the optical density at 260 nm was determined to verify the success of electroporation. Then, the suspension was centrifuged at 120,000×*g* for 90 min to remove extra PTX and ICG. Thus, three kinds of cargo-loaded EVs were obtained, namely ICG/PTX@EV, PTX@EV and ICG@EV.

### Preparation of ICG/PTX@RGE-EV

According to the approach of click chemistry, RGE-peptide was conjugated to EVs of ICG/PTX@EV by a cycloaddition reaction of sulfonyl azide. Glioma-targeting EVs were generated following two steps: (1) conjugation of the alkyne group with the protein (the phosphatidylethanolamine) on the EV membrane with a 1-Ethyl-3-(3-dimethylaminopropyl)carbodiimide/N-hydroxysuccinimide (EDC-NHS) condensation reaction and (2) conjugation of RGE-peptide with the azido group to the alkyne group by triazole linkages. The reaction mixture included 1 mL of PBS containing 35 mg sulfo-NHS and 29 mg 4-pentynoic acid, with the pH buffered to 7.4 using sodium bicarbonate. The resulting solution was stirred on ice for 1 h. Next, EDC (46 mg, 0.3 mmol) was added to the reaction mixture, and the solution was stirred on ice for 1 h. Then, 4 µL of the reaction mixture was added to 160 μg EVs in 150 μL PBS, and stirred at 20 °C for 24 h. The EVs were purified from excess reaction materials twice by ultrafiltration with a 100 kDa cut-off tube. Next, 160 μg of EVs (quantified by protein content) in 300 μL PBS, 14 μL of 0.32 M copper (II) sulfate pentahydrate, 71 μL of 1.44 M L-ascorbic acid, 32.8 μL of 0.27 M bathophenanthrolinedisulfonic acid disodium salt trihydrate, and 0.2 mg of 8.5 mM RGE-peptide (containing azido and FITC) were added, step by step, at 20 °C in a three-necked bottle. All reactants were dissolved in PBS immediately before use. Total sample volume was 425.8 μL. The reaction solution was agitated with continuous supply of nitrogen at room temperature for 4 h. To separate the EVs from the reaction mixture, samples were ultrafiltrated with a 100 KDa filter membrane at 5000×*g*, twice for 40 min. The obtained ICG/PTX@RGE-EV was stored at − 80 °C.

### Quantification of ICG/PTX@RGE-EV in RGE

To verify whether the RGE-peptide was successfully conjugated on the EVs, the following experiments were performed. Qualitative analysis was conducted using fluorescence localization imaging: (1) 10 μg/mL ICG/PTX@RGE-EV suspension was added drop-wise in the culture dish, and then FITC fluorescence (green) indicating RGE-peptide was recorded by super-resolution fluorescence microscopy; (2) 10 μg/mL ICG/PTX@RGE-EV pre-labeled with DiI (DiI was prepared by adding 10 mg DiI with 5 mL Dimethyl Sulfoxide to a concentration of 2 mg/mL; EVs were dissolved in 200 μL PBS, added with DiI storage solution, and the working concentration was adjusted to 15 μM, followed by incubation at room temperature for 30 min and washing with 10 mL PBS). This product was purified with an ultra-filter, and added drop-wise in the confocal dish, whereupon the distribution of FITC fluorescence (green) and CM-DiI fluorescence (red) was identified under confocal microscopy; (3) 10 μg/mL ICG/PTX@RGE-EVs were incubated with U251 cells for 2 h, and the fluorescence signals (CM-DiI and FITC) distributions were identified under confocal microscopy; (4) 10 μg/mL ICG/PTX@RGE-EV conjugated or mixed with RGE-peptide were incubated with U251 cells for 2 h, and the percentage of FITC-positive cells was measured by flow cytometry.

Quantitative analysis was performed using ultraviolet–visible (UV/VIS) spectrometry. Then, RGE-peptide on ICG/PTX@RGE-EV was subjected to quantitative analysis. After conjugation, free RGE-peptide was removed by ultrafiltration with Millipore (MWCO: 100KDa). The purified ICG/PTX@RGE-EV was treated with 0.1% *N*,*N*-dimethylethanolamine to avoid FITC quenching, and absorbance at 490 nm was detected by UV/VIS spectrometry. Hence, the RGE-peptide was measured as follows: (1) The absorbance values of RGE-peptide conjugated by FITC at 490 nm wavelength were measured with a spectrophotometer, and the standard curve of “absorbance value-concentration” of RGE was plotted. (2) the absorbance value of ICG/PTX@RGE-EV at 490 nm was measured, and the content/concentration of RGE in ICG/PTX@RGE-EV was calculated according to the curve. After that, the number of peptide molecules on the EVs was calculated according to the EV particle concentration determined by NTA.

### Determination of PTX and ICG contents

The contents of ICG and PTX in ICG/PTX@RGE-EV were determined by UV spectrophotometry and HPLC, respectively. The ICG content was determined as follow: (1) UV–VIS spectrophotometer (Lambda 750, PerkinElmer, UK) and FL spectrometer (LS55, PerkinElmer) were used to obtain UV–VIS spectroscopy and fluorescence emission spectrum of ICG and ICG/PTX@RGE-EV; (2) the absorbance value of ICG at 790 nm was measured to plot a standard curve of the absorbance-concentration value of ICG; (3) the absorbance value of ICG/PTX@RGE-EV at 790 nm was measured to calculate the ICG content/concentration in ICG/PTX@RGE-EVs based on the standard curve. Then, the PTX content in ICG/PTX@RGE-EV and entrapment efficiency were determined by reverse HPLC (Shimadzu LC-20AT, Japan) as follows: (1) C18 octyldecylsilane chromatographic column (250 mm × 4.6 mm × 5 μm, Thermo Fisher Scientific) was used at room temperature. A mobile phase consisting of acetonitrile: methanol and water (45: 30: 25; v/v) adjusted using 1% glacial acetic acid was delivered at a rate of 1 mL/min, and sample volumes of 20 µL were applied via an injection valve; a UV detector set at 227-nm in conjunction with spichrom software was employed for quantitation; (2) PTX was diluted first in methanol and then by mobile phase to obtain PTX samples of different concentrations to plot the calibration curve; (3) ICG/PTX@RGE-EV was lysed, diluted in mobile phase and loaded to obtain HPLC chromatograms, while PTX content and entrapment efficiency were calculated based on calibration curve.

$${\text{\% ~entrapment~efficiency~ = ~}}\frac{{{\text{Autual~amount~of~drug~loaded}}}}{{{\text{Autual~amount~of~drug~used~for~sample~preparation}}}}{\text{~}} \times {\text{~100\% }}$$

### Characterization of EVs and ICG/PTX@RGE-EV

NTA and TEM were performed to analyze particle size, zeta potential and morphology of EVs and ICG/PTX@RGE-EV. Markers of EVs, namely CD63 and CD81, were detected by Western blot analysis. NTA was performed with the ZetaView NTA (Particle Metrix, Germany) and TEM was conducted using the HT7700 TEM Hitachi. Western blot analysis procedures were as follows: EVs and ICG/PTX@RGE-EV were lysed and quantified with BCA protein assay. Next, 20 μg protein was loaded in each lane, separated by 10% sodium dodecyl sulfate–polyacrylamide gel electrophoresis (SDS-PAGE) and transferred onto the PVDF membrane. To avoid non-specific binding, PVDF membrane was blocked in 5% skimmed milk powder at room temperature for 1 h and incubated with primary antibodies to CD63 (10628D, 1: 1500, Invitrogen, Carlsbad, CA, USA), CD81 (orb388959, 1: 500, Biorbyt), Alix (CST, 92880S, 1:500), and ApoA1 (sc-30089, 1: 1000, Santa Cruz Biotechnology, USA) at 4 °C overnight. Then, the membrane was incubated with horseradish peroxidase-conjugated goat anti-mouse secondary antibody at room temperature for 30 min. The protein was visualized and photographed using Amersham ECL or ECL Plus (GE Healthcare Life Sciences).

### Stability evaluation of ICG/PTX@RGE-EV

The prepared ICG/PTX@RGE-EV were divided into 12 aliquots and stored at − 80 °C. The contents of RGE, ICG and PTX were determined on a weekly basis for 4 weeks. TEM and NTA of ICG/PTX@RGE-EV before and after 4 weeks were performed while expression of CD63, CD81 and ApoA1 was determined by Western blot analysis.

### Photothermal properties of ICG/PTX@RGE-EV

ICG, ICG/PTX@EV and ICG/PTX@RGE-EV with ICG concentration being 0, 6.25, 12.5, 25 and 50 μg/mL were exposed to 808 nm laser light at a power of 1.0 W/cm^2^ for 5 min. An infrared thermal imager (Fluke, Ti400, USA) was used to record the highest temperature of the solution every 10 s during illumination. Moreover, ICG, ICG/PTX@EV and ICG/PTX@RGE-EV with ICG concentration being 6.25 μg/mL were exposed to 808 nm laser at 0.5, 0.75, 1.0, 1.25 and 1.5 W/cm^2^ for 5 min. An infrared thermal imager was used to record the highest temperature of the solution every 10 s.

### PTX release of ICG/PTX@RGE-EV

Dynamic dialysis proved that heat released by ICG accelerated PTX release from ICG/PTX@RGE-EV under laser irradiation. When laser irradiation was at a power of 1.0 W/cm^2^, the temperature increased to approximately 60 °C. Since laser irradiation power should not be that high when living body was involved, power density of laser was set as 1.0 W/cm^2^. The sample (4 mL) was packed in a dialysis bag (MWCO: 14 kDa), submerged in 100 mL PBS, and stirred at a speed of 100 rpm at 37 °C. As for sample in the NIR group, sample was exposed to NIR light (808 nm, 1.0 W/cm^2^) at 10, 15, 20, and 24 h for 5 min. PTX concentration was determined by HPLC. The determination was performed in triplicate.

### NRP-1 expression

Western blot analysis was performed as described above using primary antibody to NRP-1 (ab81321, 1: 1000, Abcam, Cambridge, UK).

### Uptake and targeting abilities of cells in vitro

U251 and Bel-7404 cells were used to identify the targeting ability of ICG/PTX@RGE-EV in glioma cells. Cells were seeded onto the coverslip at a density of 1 × 10^4^ cells/chamber for 24 h. Bel-7404 cells were incubated with 10 μg/mL ICG/PTX@RGE-EV while U251 cells were incubated with ICG/PTX@RGE-EV, ICG/PTX@EV or ICG/PTX@RGE-EV + free RGE (pre-incubated with RGE for 5 min, blocked) for 4 h. Each chamber was rinsed twice with PBS to remove the supernatant, then immobilized by 4% paraformaldehyde solution for 15 min, and incubated with 4',6-diamidino-2-phenylindole (DAPI) solution for 10 min. Then, each well was washed twice with PBS and the cells were observed under a confocal microscope (TCS SP5, Leica).

U251 cells were implanted into a 12-well plate at a density of 5 × 10^5^ cells/well, treated as described above, rinsed twice with PBS, and collected. Cell fluorescence labeled by ICG was detected by FACSCanto (BD Biosciences, San Jose, CA, USA) for flow cytometric analysis.

### Anti-tumor action in vitro

To assess the advantage of targeting therapy and combined therapy, cell toxicity was evaluated by treating cells with chemotherapy, hyperthermia, or hyperthermia combined with chemotherapy. Cells following chemotherapy were treated with PBS, free PTX, PTX@EV, or PTX@RGE-EV. Cells following hyperthermia were treated with PBS + NIR, free ICG + NIR, ICG@EV + NIR or ICG@RGE-EV + NIR. Cells following hyperthermia combined with chemotherapy were treated with PBS, PBS + NIR, PTX@RGE-EV, ICG@RGE-EV + NIR, ICG/PTX@ EV + NIR or ICG/PTX@RGE-EV + NIR. The dose was set as 0—6.4 μg/mL relative to PTX.

As for cells without photothermal treatment, the cells were seeded into a 96-well plate at a density of 8 × 10^3^ cells/well and cultured for 24 h. Then, the cells were incubated with different reagents for 48 h and rinsed twice with PBS. Optical density of each well at 450 nm was measured using a microplate reader (Multiskan GO, Thermo Fisher Scientific) by means of a CCK8 kit (Dojindo). As for photothermal treatment, cells were seeded into a 96-well plate at a density of 8 × 10^3^ cells/well for 24 h. Then, cells were incubated with different reagents for 4 h. Each well was exposed to laser light (808 nm, 0.8 W/cm^2^) for 5 min, followed by further incubation for 44 h. Optical density of each well at 450 nm was then measured by means of a CCK8 kit.

Calcein/PI staining assay was performed as follows. Cells were seeded into a 96-well plate at a density of 3 × 10^4^ cells/well and cultured overnight, and then incubated with PBS, RGE-EV, and PTX@RGE-EV respectively for 48 h, with ICG@RGE-EV or ICG/PTX@RGE-EV for 4 h and then exposed to laser (808 nm, 0.8 W/cm^2^) for 5 min, followed by further incubation for 44 h. Subsequently, the medium in each well was discarded, whereupon 100 μL Calcein-AM (2 μmol/mL) and PI (50 μg/ mL) was supplemented for a 10-min incubation, followed by observation of staining under a confocal microscope (TCS SP5, Leica).

### Cell apoptosis

Flow cytometry was conducted as follows. U251 cells were seeded in a 6-well plate and cultured for 16–24 h, then incubated with PTX/@RGE-EV and RGE-EV (10 μg/mL PTX) for 24 h, and with ICG@RGE-EV and ICG/PTX@RGE-EV for 4 h, and then exposed to laser (808 nm, 0.8 W/cm^2^) for 5 min, followed by further incubation for 20 h. Then, cells were collected and fixed in 500 μL Annexin V binding buffer solution supplemented with 5 μL Annexin V-APC and 5 μL 7-AAD. Following incubation for 20 min at room temperature in the dark, groups of 20,000 cells were analyzed by fluorescence-activated cell sorter. PBS and PBS + NIR treatments were set as blank controls.

Western blot analysis was carried out following the aforementioned cell treatment steps. Cells were collected to extract protein, which was assayed by BCA. The protein was separated by SDS-PAGE and transferred onto a PVDF membrane, which was blocked in skimmed milk and then incubated with primary antibodies to Cleaved caspase-3 (AB3623, 1: 200, Merck Millipore, Billerica, MA, USA), Bcl-2 (3498S, 1: 1000, CST), Bax (#2774S, 1: 1000, CST), and glyceraldehyde-3-phosphate dehydrogenase (GAPDH) (#5174S, 1: 1000, CST) at 4 °C overnight, followed by 2-h incubation in the dark with goat anti-rabbit immunoglobulin G secondary antibody (KGAB015, 1: 5000, KeyGEN BioTECH). Results were visualized by chemiluminescence and electrochemiluminescence.

### Orthotopic glioma-bearing nude mouse model development

Five-week female BALB/c nude mice (The Animal Experimental Center of Zhengzhou University, Zhengzhou, China) were anesthetized using 1% pentobarbital for surgical procedures. All the operations involved in the study were approved by the animal ethics committee of The First Affiliated Hospital of Zhengzhou University. Human glioma cell line U251 (1 × 10^5^ cells/5 µL PBS) was implanted into the right striatum for obtaining xenograft tumors. Mice were anesthetized and fixed on the stereotaxic apparatus for small animals. The scalp was resected at the midline of the head and the periosteum was removed. The exposed bregma was the coordinate origin. The anterior–posterior coordinate and medio-lateral coordinate were set to zero, and the needle tip was moved to touch the skull, whereupon the depth coordinates were adjusted to zero. Next, a hole was drilled at the target point, and the needle was inserted according to a predetermined coordinate parameter. Then, 5 μL of cell suspension (1 × 10^5^ cells) was injected into the striatum at a rate of 1 μL/min, after which the needle remained in place for 3 min, followed by retraction and suturing of the scalp. Seven days later, tumor imaging was performed upon intravenous injection of fluorescein in conjunction with an in vitro imaging system (IVIS) (Caliper Life Sciences, Hopkinton, MA, USA) to confirm model establishment. The IVIS spectral imaging system was adopted to observe the growth of tumor cells. Prior to observation, each mouse was intraperitoneally injected with 150 μL of 30 mg/mL fluorescein substrate, followed by 10–25 min of intravital imaging. In detail, the IVIS spectral imaging system was rubbed by starting the *Living Image* program and clicking “initialize IVIS system” in the control panel. Then the camera was adopted for further use when it was pre-cooled to − 90 °C. Next, luminenscence mode was selected, and bioluminescence pictures were obtained by clicking “acquire”.

### Targeting ability of ICG/PTX@RGE-EV in vivo

To confirm the targeting ability of ICG/PTX@RGE-EV in vivo for localization of glioma and to determine treatment point for subsequent photothermal therapy, nude mice bearing glioma were weighed and assigned to the ICG, ICG/PTX@EV and ICG/PTX@RGE-EV groups (n = 10 nude mice/group). ICG/PTX@EV or ICG/PTX@RGE-EV containing 0.5 mg/kg ICG was intravenously injected into a tail vein, followed by observation under an in vivo imager for small animals under the same conditions at 0, 6, 12, 24, and 48 h. Major organs and tumor tissues were harvested from other nude mice at 24 h for in vitro fluorescence imaging after euthanasia.

Following fluorescence imaging in vitro, tumor tissues were fixed in 4% paraformaldehyde for 24 h, placed in 15% sucrose PBS for 24 h until sedimentation and then in 20% sucrose for 24 h until sedimentation. Brian tissues were embedded with optimal cutting temperature (OCT) compound (SAKURA, Tissue-Tek), stored at − 80 °C, and later sliced into 20-µm sections using a Cryostat (Leica, CM 1900, Wetzlar, Germany) and stained with 1 mg/mL DAPI at room temperature for 10 min. After two PBS rinses (pH 7.4), sections were immediately observed under a fluorescence microscope (Leica, DMI 4000B, Germany).

### Blood circulation and organ distribution of ICG/PTX@RGE-EV

PTX or ICG/PTX@RGE-EV was injected into nude mice bearing glioma via a tail vein at a dose of 10 mg/kg. Three mice were euthanized at 5 min, 30 min, 1, 2, 4, 8, 12 or 24 h after the drug administration (three mice were euthanized at each time point). Samples of plasma, liver, kidneys, spleen, lungs, heart and tumor were harvested and stored at − 70 °C until quantification of PTX.

PTX concentration in the plasma was determined by reverse-phase HPLC as above. Then, 50 μL of sodium acetate buffer (pH 5), 25 μL of internal standard (25 µg/mL diazepam in methanol) and 6 μL of diethyl ether were added into 250 μL of plasma samples. Sample tubes were vortexed for 2 min and centrifuged at 5000 rpm for 10 min. The upper organic layer was transferred into clean tubes and evaporated to dryness under nitrogen gas. The residue was reconstituted with 100 μL of mobile phase and an 80 μL aliquot was injected into the HPLC column. Chromatographic separation was achieved using a reverse-phase C18-Bondapak column (3.9 × 250 mm) at 58 °C. The mobile phase contained acetate buffer (0.01 M)/acetonitrile (58/42, pH 5 ± 0.1), which was eluted at a flow rate of 1.9 mL/min, and effluent was monitored at 227 nm using a UV detector. Quantitative determination was achieved by measuring the peak area ratios of the drug relative to the internal standard.

To determine the amount of PTX in different organs, tissue samples were homogenized (Heidolph, SilentCrusher S, Schwabach, Germany) with two volumes of normal saline. Approximately 250 μL of each organ homogenate was transferred into a glass tube and PTX was extracted and assayed in the same manner as described for plasma specimens.

### Anti-tumor effect of targeting combined therapy in vivo

Four days after establishing the orthotopic glioma model, photothermal therapy was performed in vivo. Six groups were set as PBS, PBS + NIR, PTX@RGE-EV (chemotherapy), ICG@RGE-EV + NIR (hyperthermia), ICG/PTX@EV + NIR (non-targeted combined therapy), and ICG/PTX@RGE-EV + NIR (targeted combined therapy). According to in vivo targeting imaging results, uptake of drugs by tumors reached the maximum at 24 h after injection. Therefore, at this time delay mice were injected with different substances intravenously, whereupon laser irradiation was performed using 808 nm laser directed at the site of tumor with a power of 0.8 W/cm^2^ for 5 min, and the infrared image was then recorded by a thermal infrared imager. The weight of mice recorded, and the growth and fluorescence intensity of tumors were obtained by IVIS with the injection of luciferin. The dose was converted to PTX, with a dose of each mouse equivalent to 10 mg/kg PTX of body weight.

The mice were euthanized by anesthesia overdose and brain tissues were collected for hematoxylin–eosin (HE) staining (C0105, Beyotime, Shanghai, China). Other nude mice were used to harvest tumor tissues for weighing, HE staining, and terminal-deoxynucleotidyl transferase mediated 2'-deoxyuridine 5'-triphosphate nick end labeling (TUNEL) staining (C1086, Beyotime). The day when mice inoculated with tumor cells was set as day 0. The number of surviving nude mice was recorded until all nude mice died to plot the survival curve.

### ICG/PTX@RGE-EV toxicity assessment

After treatment, nude mice were euthanized and dissected to collect major organs (heart, liver, spleen, lung and kidney), which were rinsed by PBS, dried, embedded with OCT (SAKURA, Tissue-Tek) and stored at − 80 °C. The tissues were later sliced into sections in Leica CM1950 and stained with a HE staining kit (C0105, Beyotime).

### Statistical analysis

All data were processed using SPSS 21.0 statistical software (IBM Corp., Armonk, NY, USA) and expressed as mean ± standard deviation. Data comparison was analyzed by independent sample *t*-test between two groups, with one-way analysis of variance (ANOVA) among multiple groups, followed by Tukey’s post hoc test. Survival curve and median survival of nude mice were analyzed by Kaplan–Meier plots. A value of *p* < 0.05 was indicative of statistical significance.

## Supplementary Information

**Additional file 1: Fig. S1.** Representative images for Western blot analysis of EV markers CD63, CD81, Alix and EV contamination index ApoA1.

**Additional file 2: Fig. S2.** Standard curves of RGE, ICG and PTX. A, Absorbance-concentration standard curve of RGE-peptide (FITC). B, Absorbance-concentration standard curve of ICG. C, HPLC peak area-concentration standard curve of PTX. Linear regression was used to calculate the regression equation. The experiment was repeated three times independently.

**Additional file 3: Fig. S3.** Temperature change of ICG, ICG/PTX@EV or ICG/PTX@RGE-EV under exposure to laser of different powers*.* A, Temperature of 300 μg/mL ICG under exposure to laser of different powers. B, Temperature of 300 μg/mL ICG/PTX@EV under exposure to laser of different powers. C, Temperature of 300 μg/mL ICG/PTX@RGE-EV under exposure to laser of different powers.

**Additional file 4: Fig. S4.** Relative positive cells detected by TUNEL staining. Tumor tissues after therapy were stained by TUNEL to calculate the percentage of positive cells. Measurement data were expressed as mean ± standard deviation. Data comparison was analyzed by one-way ANOVA among multiple groups, followed by Tukey’s post hoc test. * *p* < 0.05. The experiment was repeated three times independently.

**Additional file 5: Fig. S5.** In vitro stability of ICG/PTX@RGE-EV. A, Concentration of RGE-peptide bound on EVs. B, ICG concentration in EVs. C, PTX concentration in EVs. D, Expression of EV markers CD63, CD81 and EV contamination index ApoA1 in ICG/PTX@RGE-EV and Raw264.7 cells detected by Western blot analysis. E, Diameter and Zeta potential of EVs detected by NTA. Measurement data were expressed as mean ± standard deviation. Data comparison was analyzed by one-way ANOVA among multiple groups, followed by Tukey’s post hoc test. The experiment was repeated three times independently.

## Data Availability

The data and materials of the study can be obtained from the corresponding author upon request.

## Abbreviations

EVs: Extracellular vesicles
ICG: Indocyanine green
PTX: Paclitaxel
NIR: Near infrared
RGE: Neuropilin-1 targeting peptide
HPLC: High performance liquid chromatography
CCK-8: Cell counting kit 8
Calcein-AM/PI: Calcein Acetoxymethyl Ester/propidium iodide
TEM: Transmission electron microscope
NTA: Nanoparticle tracking analysis
UV: Ultraviolet
NRP-1: Neuropilin-1
NIR: Near infrared
UV–VIS: Ultraviolet–visible spectroscopy
PVDF: Polyvinylidene fluoride
FBS: Fetal bovine serum
FITC: Fluorescein isothiocyanate
PBS: Phosphate buffered saline
BCA: Bicinchonininc acid
EDC-NHS: 1-Ethyl-3-(3-dimethylaminopropyl) carbodiimide/*N*-hydroxysuccinimide
SDS-PAGE: Sodium dodecyl sulfate–polyacrylamide gel electrophoresis
IVIS: In vitro imaging system
